# Establishment of a Novel Risk Stratification System Integrating Clinical and Pathological Parameters for Prognostication and Clinical Decision‐Making in Early‐Stage Cervical Cancer

**DOI:** 10.1002/cam4.70394

**Published:** 2024-11-18

**Authors:** Haiying Wu, Lin Huang, Xiangtong Chen, Yi OuYang, JunYun Li, Kai Chen, Xiaodan Huang, Foping Chen, XinPing Cao

**Affiliations:** ^1^ Department of Radiation Oncology, State Key Laboratory of Oncology in South China, Collaborative Innovation Center for Cancer Medicine Sun Yat‐sen University Cancer Center Guangzhou China

**Keywords:** adjuvant therapy, cervical cancer, overall survival, postoperative risk factor, recursive partitioning analysis

## Abstract

**Background:**

Highly heterogeneity and inconsistency in terms of prognosis are widely identified for early‐stage cervical cancer (esCC). Herein, we aim to investigate for an intuitional risk stratification model for better prognostication and decision‐making in combination with clinical and pathological variables.

**Methods:**

We enrolled 2071 CC patients with preoperative biopsy‐confirmed and clinically diagnosed with FIGO stage IA‐IIA who received radical hysterectomy from 2013 to 2018. Patients were randomly assigned to the training set (*n* = 1450) and internal validation set (*n* = 621), in a ratio of 7:3. We used recursive partitioning analysis (RPA) to develop a risk stratification model and assessed the ability of discrimination and calibration of the RPA‐derived model. The performances of the model were compared with the conventional FIGO 2018 and 9th edition T or N stage classifications.

**Results:**

RPA divided patients into four risk groups with distinct survival: 5‐year OS for RPA I to IV were 98%, 95%, 85.5%, and 64.2%, respectively, in training cohort; and 99.5%, 93.2%, 85%, and 68.3% in internal validation cohort (log‐rank *p* < 0.001). Calibration curves confirmed that the RPA‐predicted survivals were in good agreement with the actual survivals. The RPA model outperformed the existing staging systems, with highest AUC for OS (training: 0.778 vs. 0.6–0.717; internal validation: 0.772 vs. 0.595–0.704; all *p* < 0.05), and C‐index for OS (training: 0.768 vs. 0.598–0.707; internal validation: 0.741 vs. 0.583–0.676; all *p* < 0.05). Importantly, there were associations between RPA groups and the efficacy of treatment regimens. No obvious discrepancy was observed among different treatment modalities in RPA I (*p* = 0.922), whereas significant survival improvements were identified in patients who received adjuvant chemoradiotherapy in RPA II–IV (*p* value were 0.028, 0.036, and 0.024, respectively).

**Conclusion:**

We presented a validated novel clinicopathological risk stratification signature for robust prognostication of esCC, which may be used for streamlining treatment strategies.

## 1 Introduction

The International Federation of Gynecology and Obstetrics (FIGO) and the American Joint Committee on Cancer (AJCC)/Union for International Cancer Control (UICC) TNM staging system are generally accepted instruments for formulating efficacious therapeutic strategies and predicting prognositication [1, 2].

The FIGO systems were updated in 2018 with the incorporation of lymph node status and more granular tumor dimensions, with an emphasis on imaging findings rather than focusing solely on anatomical information, thus altering the patient's risk profile. Nevertheless, current staging systems based on gynecological examination and imaging signs are constrained by inherent limitations in terms of sensitivity and specificity, as well as personal experience, introducing tumor heterogeneity and resulting in inaccurate prognostication. Lee et al. found that the consistency index for assessing 5‐year survival after surgery for early‐stage cervical cancer (esCC) based on FIGO staging alone was only 0.54 [3]. A previous study reached the conclusion that stage alone accounted for approximately 60% of the prognostic information, with the other factors accounting for the remainder in local advanced cervical cancer [4].

Currently, the guidelines of National Comprehensive Cancer Network (NCCN) are mainly based on FIGO staging systems to help guide treatment decisions [5]. Radical hysterectomy is the preferred treatment for esCC. It can be reasonably concluded that postoperative risk factors exert a non‐negligible value on risk stratification and prognostic prediction for esCC. However, the controversy persists surrounding the risk factors included in the current guideline, referring to the high‐risk factors (lymph node metastasis (LNM), positive resection margins, and parametrium infiltration), and intermediate risk factors included in the “Sedlis” criteria (stromal invasion, lymphovascular space invasion, and large tumor size), and there is no consensus on the optimal selection of postoperative regimen [6, 7].

Current factors outlined in the guidelines are deemed inadequate and limited, which has drawn researchers' attention to several underappreciated factors that we had previously overlooked. Levinson points out that the threshold of “Sedlis” is quite high and potentially excludes certain CC patients from consideration of adjuvant therapy [8]. Cibula proposes that the tumor‐free distance could be recognized as a novel negative prognostic indicator to serve as an intermediate‐risk factor, while Chen suggests that perineural invasion might be acknowledged as a new high‐risk factor for cervical cancer [9, 10]. In addition, corpus invasion, as well as degree of differentiation may also contribute to tumor malignancy and survival outcome [11, 12, 13, 14]. Prior publications have been conducted to build new models, but there are certain limitations to clinical application. A nomogram model only considered the intermediate risk factors and included treatment‐related variables [15]. A multi‐center study presented a model incorporating demographic and clinicopathological factors to predict 5‐year overall survival after surgery for stage IB–IIA CC; the model, however, only considered patients who received radiotherapy after surgery [3]. Therefore, it is urgent to develop a comprehensive and robust classification model to alter current postoperative risk stratification in esCC.

As yet no studies incorporating postoperative clinicopathological parameters and applying recursive partitioning analysis (RPA) have been developed to construct a model for esCC patients. Here, we provide a retrospective analysis to explore the interactions and synergy effect among these parameters based on a large dataset, thus proposing a novel risk stratification signature for robust prognostication and guiding clinical decision‐making for esCC patients.

## 2 Materials and Methods

### 2.1 Patient Selection

In this retrospective study, we initially enrolled 2892 women with biopsy‐proven and clinically diagnosed as cervical cancer with stage IA–IIA preoperatively according to FIGO 2009, who received surgical procedure at the Department of Gynecology of Sun Yat‐sen University Cancer Center, from January 2013 to December 2018. All patients underwent routine preoperative assessments, including gynecological examination, imaging, and hematology examinations. Perioperative factors, postoperative detailed treatment regimens, and follow‐up data were collected from a panoramic database within “Sun Yat‐Sen University Cancer Center‐Intelligence Platform for Cancer Research” [16]. The inclusion criteria were as follows: (1) histologically diagnosed as squamous carcinoma, adenocarcinoma, and adenosquamous carcinoma (SCC, AC, and ASC). (2) complete surgical and pathological records. (3) Karnofsky performance status score ≥ 70. Details of exclusion criteria are shown in Figure S1. After excluding the non‐conforming samples, 2071 samples were used for subsequent analysis. These cases were restaged by two radiation oncologists specializing in gynecological oncology using the FIGO 2018 staging system. The dataset was randomly partitioned into a training set (*n* = 1450) and an internal validation set (*n* = 621) in a 7:3 ratio.

### 2.2 Treatment and Follow‐Up

In general, radical hysterectomy + pelvic lymph node dissection or sentinel lymph node biopsy is recommended for esCC, with postoperative adjuvant therapy as appropriate for those with high or intermediate risk factors. Neoadjuvant chemotherapy (NACT) and adjuvant chemotherapy (ACT) regimen consisted of paclitaxel plus platinum and was delivered triweekly for one to four cycles. Intensity‐modulated radiotherapy (IMRT) is the usual technique, with a prescribed dose for the clinical target volume (CTV) of 45–50 Gy and gross tumor volume (GTV) of 60 Gy in 25 fractions. Brachytherapy is employed as a boost to external beam radiation therapy (EBRT), particularly in cases of positive and close vaginal surgical margins or vaginal intraepithelial neoplasia (VAIN). The concurrent chemoradiotherapy (CCRT) regimens comprised single‐agent platinum or a double‐agent paclitaxel plus platinum regimen.

Upon completion of treatment, patients were followed up by outpatient review and telephone period. We defined the time from the diagnosis to all‐cause death or to the last follow‐up as overall survival (OS), which is the primary endpoint. Progression‐free survival (PFS), to recurrence, metastasis, death, or the last follow‐up. Locoregional relapse‐free survival (LRRFS), to first relapse in the central or lateral pelvic region, and lesions in retroperitoneal or pelvic lymph nodes; while distant metastasis‐free survival (DMFS), to recurrences in distant organs or non‐draining lymph nodes.

### 2.3 Risk Model Development

Clinicopathological parameters were first analyzed by Cox regression analysis. Next, least absolute shrinkage and selection operator (LASSO) regression was further carried out to screen the optimal prognostic factors.

RPA [17] was used to group patients with similar OS based on covariates splits and combinations. The most significant variable with the best‐split point is determined after multiple attempts for each variable, thus ensuring the minimum Gini value and the best internal consistency and purity of two subgroups obtained from each split [18]. And then, keep splitting down until a predetermined criterion is reached. After recursive analysis, subgroups with similar survival rates were combined into a single refined group.

### 2.4 Statistics

Follow‐up time was calculated using the reverse Kaplan–Meier method. Chi‐squared or Fisher's exact test was used to compare differences in categorical variable composition ratios between two or more groups. Kaplan–Meier (KM) analysis was done to present time‐to‐death or progression data and comparison between survival curves were performed by means of a log‐rank test. The receiver operating characteristic (ROC), time‐dependent area under the curve (tAUC), and Harrell's C‐index were used to evaluate the discrimination ability of the models. Decision curve analysis (DCA) was performed to assess clinical effectiveness using the R package “ggdca.” Hazard ratio (HR) is a relative indicator calculated by the COX proportional risk regression model to quantify the disparity among treatment modalities. R software (version 4.2.0) was used for statistical analyses. A two‐sided *p <* 0.05 was considered significant.

## 3 Results

### 3.1 Clinical Features, Failure Patterns, and Survival

Patients' clinical characteristics in the total, training, and internal validation cohort are presented in Table 1. The median follow‐up time for the overall dataset was 74.9 months (95% CI, 73.2–76.6) and the median age was 49 years (interquartile range, 43–55 years). During the follow‐up period, we recorded detailed failure patterns (Table S1). The 5‐year OS and PFS were 90.8%, 87.4% in the training cohort, and 91.6%, 89.2% in the internal validation cohort.

**TABLE 1:** Baseline characteristics.

Characteristic	Total cohort (*N* = 2071)	Training cohort (*N* = 1450)	Internal validation cohort (*N* = 621)	*p*
Age, *n* (%)				0.999
< 49	982 (47.4)	687 (47.4)	295 (47.5)	
≥ 49	1089 (52.6)	763 (52.6)	326 (52.5)	
Histology, *n* (%)				0.318
SCC	1723 (83.2)	1194 (82.3)	529 (85.2)	
AC	284 (13.7)	204 (14.1)	80 (12.9)	
ASC	64 (3.09)	52 (3.59)	12 (1.93)	
Differentiation, *n* (%)				0.821
Low	1060 (51.2)	755 (52.1)	305 (49.1)	
Medium	837 (40.4)	576 (39.7)	261 (42.0)	
High	174 (8.40)	119 (8.21)	55 (8.86)	
Lymph node metastasis, *n* (%)				1.000
Negative	1647 (79.5)	1153 (79.5)	494 (79.5)	
Positive	424 (20.5)	297 (20.5)	127 (20.5)	
Parametrium, *n* (%)				0.465
Negative	1980 (95.6)	1381 (95.2)	599 (96.5)	
Positive	91 (4.39)	69 (4.76)	22 (3.54)	
Resection margin, *n* (%)				0.963
Negative	1714 (82.8)	1205 (83.1)	509 (82.0)	
Positive	156 (7.53)	109 (7.52)	47 (7.57)	
VAIN	201 (9.71)	136 (9.38)	65 (10.5)	
Tumor size, *n* (%)				0.955
≤ 2	595 (28.7)	411 (28.3)	183 (29.5)	
> 2, ≤ 4	932 (45.0)	661 (45.6)	271 (43.6)	
> 4	545 (26.3)	378 (26.1)	167 (26.9)	
Stromal invasion, *n* (%)				0.925
< 1/2	874 (42.2)	616 (42.5)	258 (41.5)	
≥ 1/2	1197 (57.8)	834 (57.5)	363 (58.5)	
LVSI, *n* (%)				0.908
Negative	1209 (58.4)	851 (58.7)	358 (57.6)	
Positive	862 (41.6)	599 (41.3)	263 (42.4)	
Perineural involvement, *n* (%)				0.555
Negative	1856 (89.7)	1294 (89.2)	564 (90.8)	
Positive	213 (10.3)	156 (10.8)	57 (9.18)	
Vaginal invasion, *n* (%)				0.935
Negative	1543 (74.5)	1077 (74.3)	466 (75.0)	
Positive	528 (25.5)	373 (25.7)	155 (25.0)	
Corpus uterine invasion, *n* (%)				0.681
Negative	1790 (86.4)	1247 (86.0)	543 (87.4)	
Positive	281 (13.6)	203 (14.0)	78 (12.6)	
FIGO 2018, *n* (%)				1.000
IA1	157 (7.58)	107 (7.38)	50 (8.05)	
IA2	44 (2.12)	30 (2.07)	14 (2.25)	
IB1	325 (15.7)	222 (15.3)	103 (16.6)	
IB2	515 (24.9)	368 (25.4)	147 (23.7)	
IB3	196 (9.46)	135 (9.31)	61 (9.82)	
IIA1	187 (9.03)	134 (9.24)	53 (8.53)	
IIA2	191 (9.22)	132 (9.10)	59 (9.5)	
IIB	32 (1.55)	25 (1.72)	7 (1.13)	
IIIC1p	403 (19.5)	281 (19.4)	122 (19.6)	
IIIC2p	21 (1.01)	16 (1.10)	5 (0.81)	
T category, *n* (%)				0.685
T1	1497 (72.3)	1040 (71.7)	457 (73.6)	
T2	574 (27.7)	410 (28.3)	164 (26.4)	
N category, *n* (%)				0.983
N0	1647 (79.5)	1153 (79.5)	494 (79.5)	
N1	403 (19.5)	281 (19.4)	122 (19.6)	
N2	21 (1.01)	16 (1.10)	5 (0.81)	
Rates of survival outcomes, %				
5‐year OS	91.0	90.8	91.6	0.998
5‐year PFS	88.0	87.4	89.2	0.633
5‐year LRRFS	91.6	90.9	93.2	0.273
5‐year DMFS	93.0	93.3	92.6	0.846

Abbreviations: AC, adenocarcinoma; ASC, adenosquamous carcinoma; DMFS, distant metastasis‐free survival; LRRFS, locoregional relapse‐free survival; LVSI, lymph‐vascular space invasion; OS, overall survival; PFS, progression‐free survival; SCC, squamous carcinoma; VAIN, vaginal intraepithelial neoplasia.

### 3.2 Prognostic Value of Clinicopathological Characteristics

Survival analysis was carried out for each of the 12 factors in the training cohort, which indicated the prognostic values of those characteristics except for age (log‐rank *p*‐values: Age, *p* = 0.081; others, *p* ≤ 0.01) (Figure S2A **–** S2L). Univariate Cox regression analysis identified 11 parameters, which were entered into multivariate Cox regression. Considering the presence of confounding variables and multicollinearity, some variables that were considered clinically relevant showed no significance in multivariate analysis (Table S2). We turn to LASSO regression analysis, screening 11 variables for subsequent modeling (Figure S2M **–** S2N), which encompassed: histology, grade of differentiation, LNM, parametrium infiltration, resection margin, lymph‐vascular space invasion (LVSI), stromal invasion, tumor size, perineural involvement, corpus uterine invasion, and vaginal invasion. The relevant histological images are presented in Figure S3.

### 3.3 Development and Validation of RPA Staging Model

**FIGURE 1:**
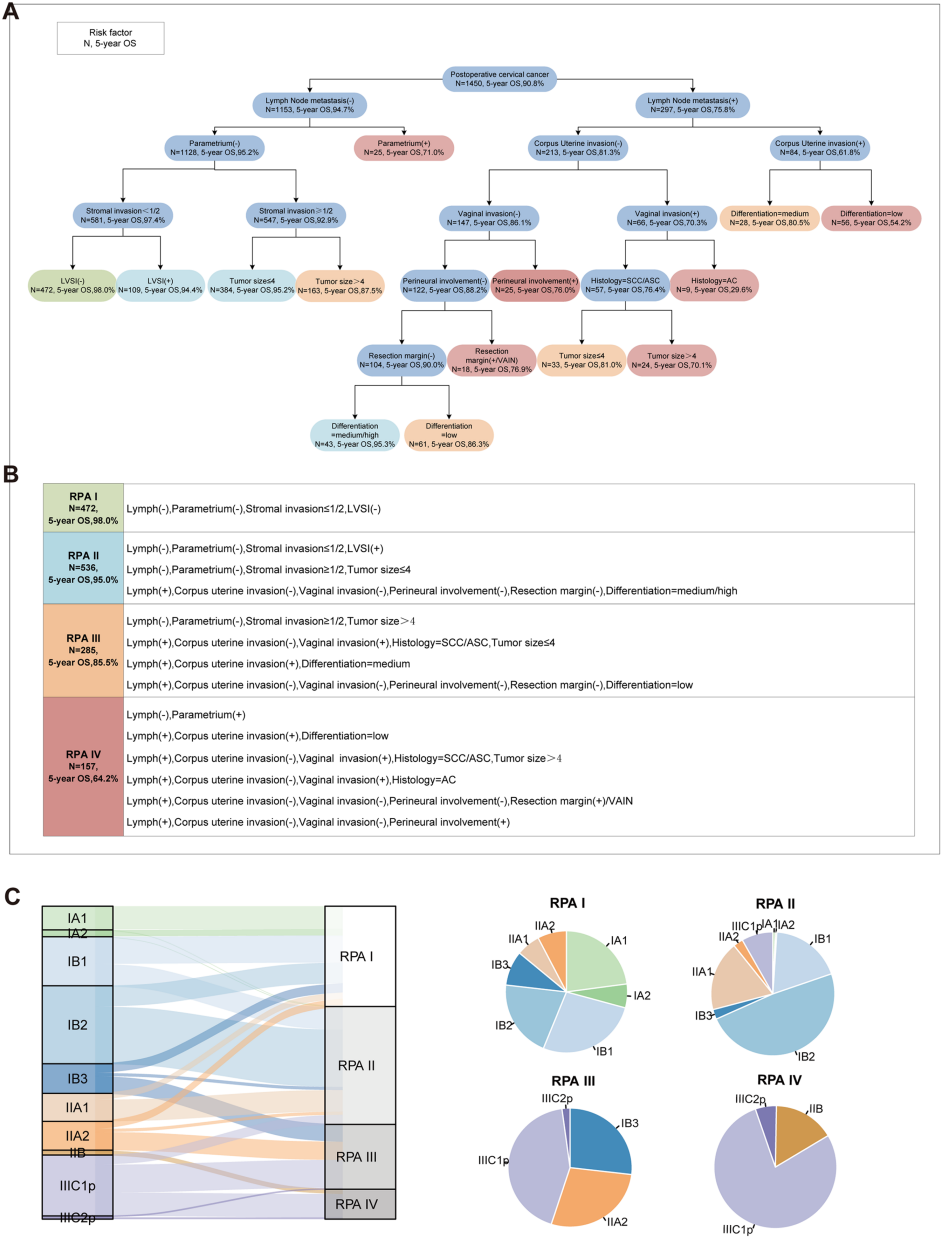
Recursive Partitioning Tree and association between FIGO 2018 and proposed RPA group. (A) Recursive partitioning tree showed the process of patients partitioning. The stopping criteria were set to a minsplit = 9, the minimum number of observations that must exist in a node in order for a split to be attempted. (B) Detailed classifications of the four RPA groups. (C) Stage migration from FIGO 2018 to RPA stage and pie plots elucidate the distribution of stages within the four groups. AC, adenocarcinoma; ASC, adenosquamous carcinoma; LVSI, lymph‐vascular space invasion; SCC, squamous carcinoma; VAIN, vaginal intraepithelial neoplasia.

In our RPA tree, the algorithm stratified patients into 14 groups, with a range of 5‐year OS from 29.6% to 98.0% (Figure 1A). Patients in diverse subgroups were grouped into four RPA groups in accordance with similar survival rates. The overall survival rates were calculated as follows: RPA I at 98.0%, RPA II at 95.0%, RPA III at 85.5%, and RPA IV at 64.2%. The detailed classifications of the four groups are listed in Figure 1B. Furthermore, we elucidated the distribution of stages within the four groups (Figure 1C). The subgroups analysis illustrated clear distinction for all parameters among four subgroups (*p* < 0.001 for all, Table S3).

**FIGURE 2:**
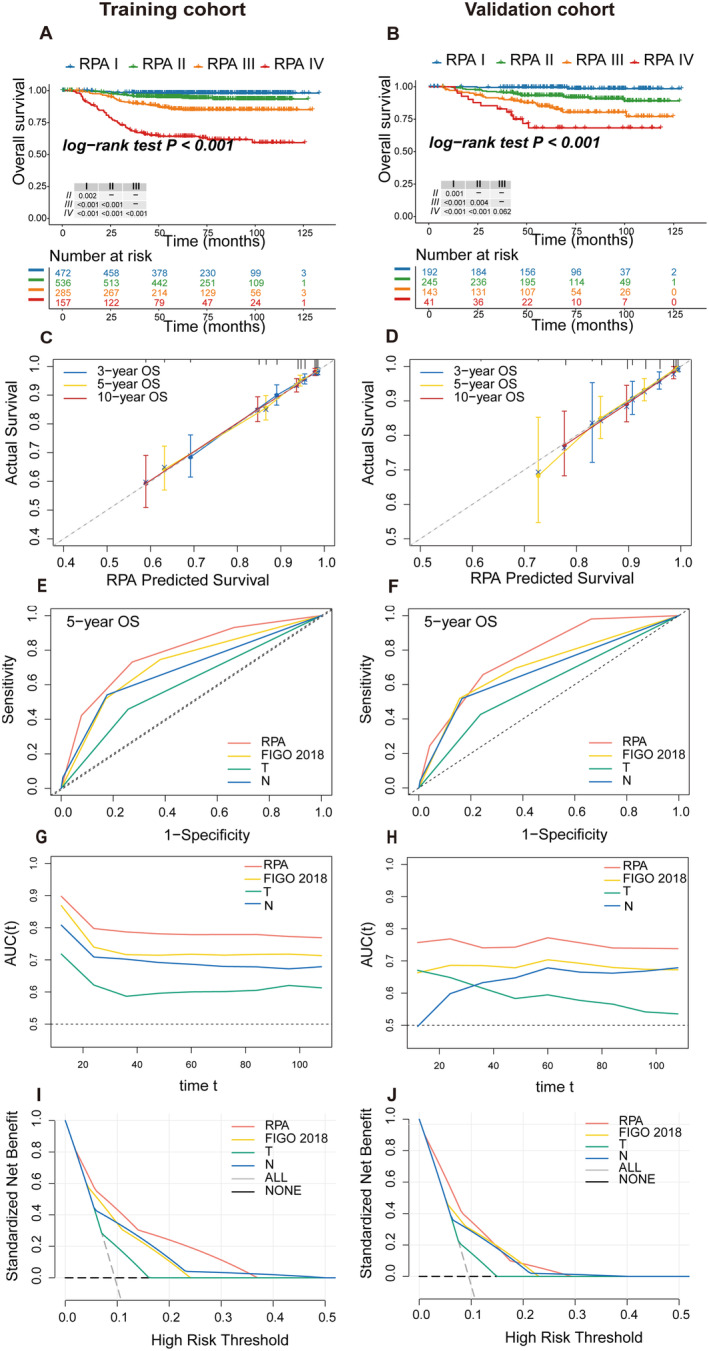
Kaplan–Meier curves among RPA groups and comparisons of the new RPA stage and other existing staging systems in terms of overall survival in training and internal validation cohorts. (A, B) Kaplan–Meier curves for training cohort (A) and validation cohort (B). (C, D) Calibration plot predicted the 3, 5, and 10‐year overall survival for training cohort (C) and validation cohort (D). (E, F) Receiver operating curves for training cohort (E) and validation cohort (F). (G, H) Time‐dependent area under curves (AUC) for training cohort (G) and validation cohort (H). (I, J) Decision curve analysis for training cohort (I) and validation cohort (J); the “All” curve represents the net benefit when all subjects are intervened, while the “NONE” curve represents no patients receiving clinical intervention. AUC, the area under the receiver operating characteristic curve; OS, overall survival; RPA, recursive partitioning analyze.

In terms of KM curves, patients in higher‐grade group exhibited a less favorable prognosis than lower‐grade group, and the result of pairwise overall survival analysis also revealed significant distinction in training cohort and internal validation cohort (5‐year OS: training cohort: I = 98%, II = 95%, III = 85.5%, IV = 64.2%; internal validation cohort: I = 99.5%, II = 93.2%, III = 85%, IV = 68.3%; both *p* < *0*.001) (Table S4 and Figure 2A,B). The calibration curves demonstrated good consistency between actual observation and the prediction by RPA model (Figure 2C,D). Moreover, similar findings were observed in progression survival prediction (Figure S4A **–** S4D).

### 3.4 Comparison of Performances Among Models

Meanwhile, we compared the new RPA model and other existing staging systems. ROC curves were constructed to explore whether the model was accurate in prognostic evaluation and areas under the ROC curve (AUC) at 5 years are illustrated (Figure 2E,F, Tables 2 and S5). In the training cohort, RPA risk stratification had the highest AUC for OS (0.778 vs. 0.6–0.717), PFS (0.715 vs. 0.584–0.668), and DMFS (0.753 vs. 0.578–0.689) (*p* ≤ 0.007 for all comparisons) (Table 2). In the internal validation cohort, the RPA model performed better for OS (0.772 vs. 0.595–0.704), and LRRFS (0.76 vs. 0.59–0.71), while the AUC did not differentiate between the RPA model and FIGO 2018 for PFS and DMFS (Table S5). Furthermore, the RPA model's time‐dependent AUC between 12 and 108 months was consistently higher than that of the other models (Figure 2G,H). DCA analyses demonstrated that the RPA model possessed the highest net benefit, suggesting high clinical applicability (Figure 2I,J). The C‐index for overall survival was 0.768 (95% CI, 0.729–0.807) in the RPA model, which was significantly higher than that for FIGO 2018 and T or N category (Table 2). Similar results emerged in terms of PFS and DMFS (Table 2). The internal validation cohort yielded similar tendency as well (Table S5).

**TABLE 2:** The comparisons of AUC and C‐index of RPA model with the FIGO 2018/9th edition T category/N category in training cohort.

	OS			PFS			LRRFS			DMFS		
	AUC/C‐index	95% CI	*p*	AUC/C‐index	95% CI	*p*	AUC/C‐index	95% CI	*p*	AUC/C‐index	95% CI	*p*
AUC												
RPA	0.778	0.737–0.82	Ref	0.715	0.673–0.757	Ref	0.693	0.643–0.745	Ref	0.753	0.700–0.805	Ref
FIGO 2018	0.717	0.672–0.763	0.002	0.668	0.626–0.71	0.007	0.659	0.611–0.708	0.105	0.689	0.634–0.744	0.002
T	0.6	0.555–0.646	< 0.001	0.584	0.545–0.623	< 0.001	0.587	0.541–0.632	< 0.001	0.578	0.526–0.630	< 0.001
N	0.686	0.641–0.732	< 0.001	0.639	0.6–0.678	< 0.001	0.629	0.584–0.674	0.002	0.661	0.608–0.715	< 0.001
C‐index												
RPA	0.768	0.729–0.807	Ref	0.707	0.668–0.746	Ref	0.686	0.638–0.734	Ref	0.743	0.694–0.792	Ref
FIGO 2018	0.707	0.665–0.749	< 0.001	0.663	0.625–0.701	0.006	0.652	0.607–0.697	0.093	0.686	0.634–0.738	0.005
T	0.598	0.557–0.639	< 0.001	0.58	0.544–0.616	< 0.001	0.58	0.538–0.622	< 0.001	0.574	0.525–0.623	< 0.001
N	0.676	0.635–0.717	< 0.001	0.634	0.598–0.67	< 0.001	0.625	0.583–0.667	0.001	0.658	0.608–0.708	< 0.001

Abbreviations: AUC, the area under the receiver operating characteristic curve; CI, confidence interval; C‐index, concordance index; DMFS, distant metastasis‐free survival; LRRFS, locoregional relapse‐free‐survival; OS, overall survival; PFS, progression‐free survival.

NACT is not considered a standard treatment internationally. To avoid its impact on the generalizability of the model, we excluded all patients who received NACT and then conducted the analysis and comparisons which presented decent results (Figures S5 and S6, Tables S6 and S7).

### 3.5 Association Between RPA Groups and Treatment Modalities

**FIGURE 3:**
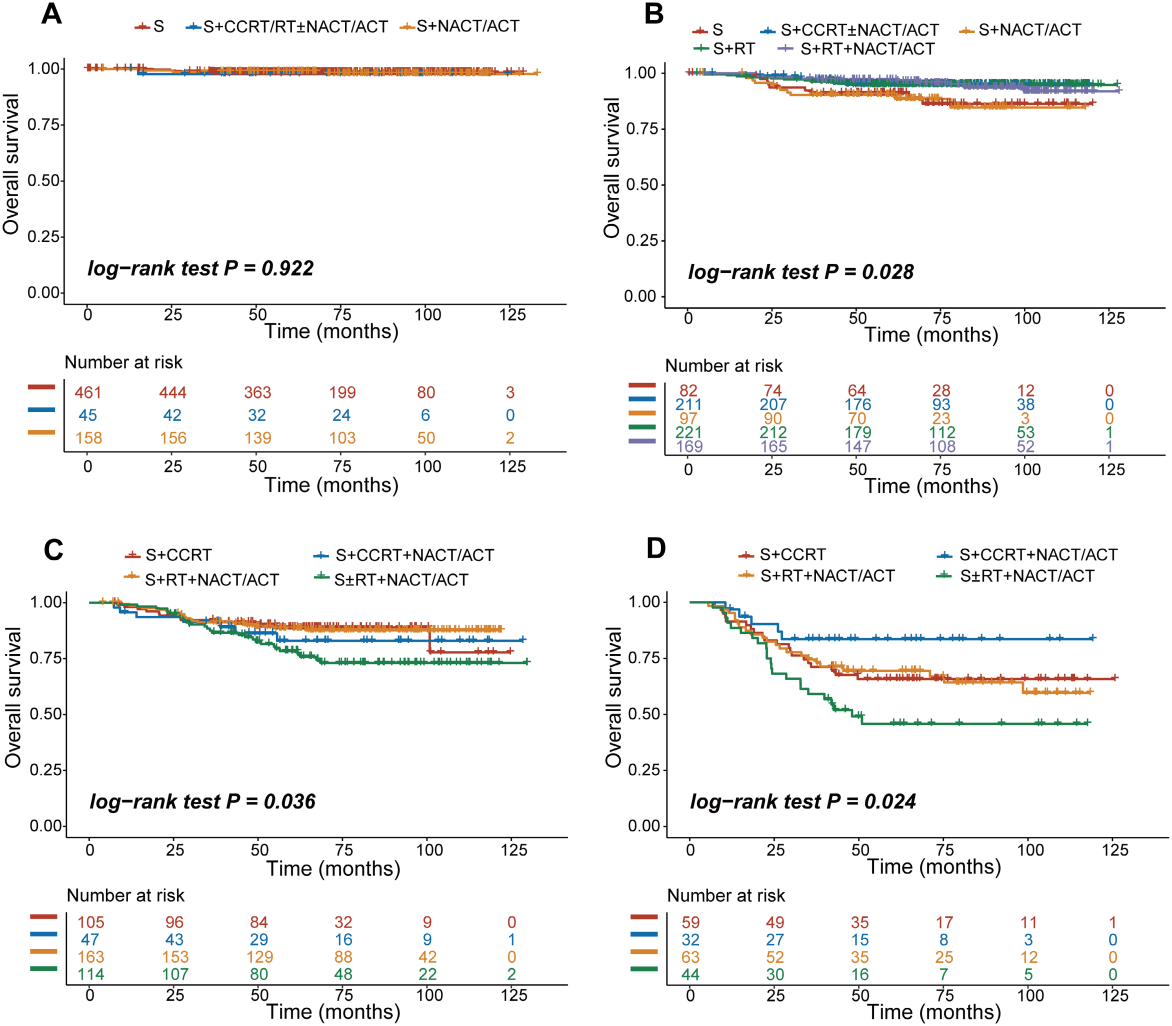
The Kaplan–Meier curves for overall survival according to different treatment protocols in RPA I–IV group (A–D). ACT, adjuvant chemotherapy; CCRT, concurrent chemoradiotherapy; NACT, neoadjuvant chemotherapy; RT, radiotherapy; S, surgery.

We analyzed the treatment patterns and calculated hazard ratios within each group in the RPA model. For RPA I group, there were no significant differences among the treatment modes (*p* = 0.922) (Figure 3A and Table S8). For those patients categorized as RPA II group, surgery alone or S + NACT/ACT exhibited prominently poorer overall survival compared with those receiving post‐operative adjuvant modes containing radiotherapy (*p* = 0.028, Figure 3B). The outcome of S + NACT/ACT were closer to surgery alone (HR_OS_ 1.084, 95% CI: 0.449–2.618, *p* = 0.857), whereas S + RT, S + CCRT ± NACT/ACT, and S + RT + NACT/ACT had much better outcomes (Table S8). Given the relatively small number of patients treated with surgery alone or postoperative radiotherapy or chemotherapy alone in RPA III/IV group, we grouped these regimens together. Within refined group III, the prognosis of postoperative CCRT or RT + NACT/ACT preceded that of S ± RT/NACT/ACT. With regard to RPA IV, we observed that S + CCRT + NACT/ACT was significantly superior to surgery, although not all pairwise comparisons reached the significance levels due to the limited sample size (Figure 3D and Table S8).

On the strength of these findings, we put forward corresponding treatment recommendations, where NCCN risk stratification, actual treatment modalities, and failure patterns were simultaneously presented (Figure S7).

## 4 Discussion

Historically, traditional staging systems have been adopted to summarize a patient's disease and prognosis, which may not accurately reflect the severity of malignancy. Our clinical practice confirm that the apparent heterogeneity of outcomes remained in esCC. We identified four unique subgroups with markedly different survival outcomes by integrating clinically relevant and available variables which have been proven to be associated with prognosis and may create a cumulative effect for postoperative esCC patients. It is clear that our model performed more satisfactorily compared to the existing models in terms of outcome prediction, risk discrimination and degree of calibration.

We have stressed the dominance and comprehensiveness of this RPA‐proposed signature by means of RPA, as it may offer valuable information for patients and clinical decision makers. Postoperative risk stratification using RPA algorithm have been reported in other neoplasms. For example, surgical colorectal adenocarcinoma patients were categorized as four risk groups based on six clinicopathological factors [19]. Pathologic nodal classification was developed for prostate cancer and cutaneous melanoma [20, 21]. In addition, RPA was performed to evaluate which part of patients would benefit from postoperative radiotherapy in lymphoepithelial carcinoma of salivary gland [22].

At the top of the RPA segmentation, LNM is the most meaningful indicator, which was in line with the previous studies and clinical practice [23, 24, 25]. Parametrium infiltration and corpus invasion were the second most influential factors in the lymph node‐positive and negative groups, respectively. A subclassification in RPA IV emphasized that parametrium infiltration was an independent malignant factor in the absence of LNM, which is in agreement with the study indicating no significant prognosis disparity between IIB and IIIC‐(T1‐T2b) [26]. Parametrium infiltration is often accompanied by large tumor size, poorer differentiation, LVSI, and perineural involvement in cervical cancer [27, 28]. Corpus uterine invasion stands for the upper boundary of tumor extension and is an independent predictor of ovarian metastasis [29]. According to the findings of Narayan, the extension of tumor in the uterine body was the most important predictor of LNM [30].

In FIGO 2018, patients are classified as stage IIIC as long as lymph node metastases occur, which indicates a high degree of malignancy. Contrary to our previous perceptions, there was a small subclassification in the RPA II group: lymph node metastases with no corpus invasion, no vaginal or perineural involvement, negative margins, and a high or moderate degree of differentiation, which suggested that this patient population would not suffer inferior prognosis partly due to relatively fewer positive lymph nodes and smaller diameter of LNM. Further studies are required to confirm the feasibility of de‐escalation treatment protocols in this patient population. In conclusion, LN status alone is insufficient to summarize all features and explain the heterogeneity in esCC. Duan et al. restaged IIIC according to local tumor size to ensure better management for CC patients in stage IIIC [26]. Other investigators also insisted on the view that great importance should be attached not only to the lymph node metastases but also to local tumor factors [31, 32].

The RPA model attenuated the importance of status of resection margin, which differs from the global guideline. The positive resection margin was deemed less pronounced due to the combination of other factors. Otherwise, brachytherapy is used to be a boost to EBRT, especially for those with positive or vain surgical margins, thereby improving local control and survival [33, 34]. A high utilization of vaginal brachytherapy and advances in surgical technique and diagnostic imaging technology are major contributors to improved outcomes.

Moreover, adenocarcinoma has a worse prognosis with a higher risk grade for RPA, which was also confirmed in our study. Patients with adenocarcinoma are highly invasive and more prone to develop high‐grade metastases, multisite metastases, and bilateral metastases [35, 36]. Likewise, specific subcategories with poor differentiation were up‐classified to RPA III or IV. Although the aforementioned parameters have not been included in current staging systems, past evidence has provided insight into their prognostic values and gives us clues to ascertain new detailed staging systems for future postoperative treatment guidance.

Regarding the treatment mortality, in those patients with negative postoperative lymph nodes, no parametrium infiltration, less than 1/2 stromal invasion, and no LVSI, surgical alone is sufficient to achieve the goal of the low incidence of mortality and recurrence. Furthermore, our findings highlight the power of radiotherapy in the intermediate‐risk group. Spared the toxicities of chemotherapy, radiation alone is sufficient for the RPA II group. It is also noteworthy that chemotherapy combined with radiotherapy may provide a better clinical curative effect in higher‐risk groups, of which postoperative CCRT + NACT/ACT was recommended for patients in the RPA IV group, and CCRT or S + RT + NACT/ACT for RPA III. However, CCRT + NACT/ACT failed to demonstrate an advantage in RPA III group owing to a limited sample size. Due to the redundancy of the data and the inevitable bias during the treatment process, it is difficult to determine the optimal treatment for each category of patients, but our risk signature can serve to streamline treatments. Clinical trials dedicated to specific subclassification are expected to be conducted to explore targeted therapies.

NACT may confound the pathological assessment of surgical resection specimens, thereby complicating the assessment of the necessity for ACT following surgery. In our study, tumor size and vaginal invasion were assessed based on pre‐treatment evaluations, and lymph nodes or stromal with “post‐treatment changes” in the pathological reports were considered positive and documented. These baseline characteristics reflect the severity of the disease before the initiation of neoadjuvant therapy. The model maintained robust performance after excluding patients who received NACT. For this particular patient cohort, the extent of tumor regression is a critical determinant of prognosis. Zannoni proposed a tumor regression system that captures the early treatment response within the context of the therapeutic regimen, thereby facilitating the adjustment of postoperative therapy and clinical decision‐making [37].

There are several restrictions in our study. For instance, we have not adequately considered the prognostic implications of the different operation modality, for a proportion of patients underwent laparoscopic surgery prior to the publication of the results of the Laparoscopic Approach to Cervical Cancer (LACC) [38]. Data regarding stromal invasion were not divided into superficial 1/3, middle 1/3, and deep 1/3 due to the non‐updated pathology reports, which is not consistent with the “Sedlis” criteria. Additionally, our study was a retrospective observational study conducted at a single center. Further external validation and prospective studies are required to verify our findings.

## 5 Conclusion

To summarize, our results cast a new light on risk stratification within postoperative esCC patients. The RPA model allows for a more accurate categorization of patients with postoperative esCC. More importantly, it is more intuitive and practical than traditional predictive models, potentially facilitating broader clinical adoption. Furthermore, our findings suggested the possibility of de‐escalation treatment protocols for some specific subgroups and up‐escalation for another part. We encourage clinicians to evaluate our proposed RPA signature, as it can better reflect heterogeneity of outcomes and provide treatment strategies for postoperative esCC patients.

## Author Contributions

**Haiying Wu:** conceptualization (equal), data curation (equal), formal analysis (equal), funding acquisition (equal), investigation (equal), methodology (equal), project administration (equal), software (equal), validation (equal), visualization (lead), writing – original draft (lead), writing – review and editing (lead). **Lin Huang:** conceptualization (equal), data curation (equal), formal analysis (equal), investigation (equal), methodology (equal), project administration (equal), software (equal), validation (equal), visualization (equal), writing – original draft (lead), writing – review and editing (equal). **Xiangtong Chen:** conceptualization (equal), data curation (equal), formal analysis (equal), investigation (equal), methodology (equal), project administration (equal), software (equal), visualization (equal), writing – original draft (equal), writing – review and editing (equal). **Yi OuYang:** conceptualization (equal), data curation (equal), formal analysis (equal), investigation (equal), methodology (equal), project administration (equal), visualization (equal), writing – original draft (equal), writing – review and editing (equal). **JunYun Li:** conceptualization (supporting), data curation (supporting), investigation (supporting), resources (equal), writing – original draft (supporting), writing – review and editing (supporting). **Kai Chen:** data curation (supporting), formal analysis (supporting), investigation (supporting), visualization (supporting), writing – original draft (supporting), writing – review and editing (supporting). **Xiaodan Huang:** data curation (equal), methodology (supporting), software (equal), writing – review and editing (supporting). **Foping Chen:** conceptualization (lead), data curation (lead), formal analysis (equal), funding acquisition (equal), investigation (equal), resources (lead), software (equal), supervision (lead), writing – original draft (equal), writing – review and editing (equal). **XinPing Cao:** conceptualization (lead), data curation (lead), funding acquisition (lead), investigation (equal), resources (lead), software (equal), supervision (lead), writing – original draft (equal), writing – review and editing (equal).

## Ethics Statement

The study was approved by the Ethics Committee of Sun Yat‐sen University Cancer Center. Written informed consent was waived since no personal information was exposed and no intervention in patient treatment.

## Conflicts of Interest

The authors declare no conflicts of interest.

## Supporting information


Data S1.

## Data Availability

The key raw data was uploaded onto the Research Data Deposit public platform (www.researchdata.org.cn).
