# Supplementary material for: Establishment of a Novel Risk Stratification System Integrating Clinical and Pathological Parameters for Prognostication and Clinical Decision‐Making in Early‐Stage Cervical Cancer
Source: Cancer Med. 2024 Nov 18;13(22):e70394. doi: 10.1002/cam4.70394 (PMC11571237; doi:10.1002/cam4.70394)
Supplement: Supplementary file 1 — Data S1. [file CAM4-13-e70394-s001.docx]

Supporting Information

Table S1. Recurrence and distant metastasis patterns in the overall, training, and internal validation cohort.

|  | Overall cohort  (N =2071) | Training cohort  (N = 1450) | Internal validation cohort  (N=621) |
| --- | --- | --- | --- |
| Locoregional | 178 (8.59%) | 134 (9.24%) | 44(7.09%) |
| Local | 89 (4.30%) | 67 (4.62%) | 22 (3.54%) |
| Regional | 50 (2.41%) | 36 (2.48%) | 14 (2.25%) |
| Both | 39 (1.88%) | 31 (2.14%) | 8 (1.29%) |
| Distant | 145 (7%) | 98(6.76%) | 47(7.57%) |
| Lung | 35 (1.69%) | 24 (1.66%) | 11 (1.77%) |
| Bone | 8 (0.39%) | 6 (0.41%) | 2 (0.32%) |
| Liver | 10 (0.48%) | 7 (0.48%) | 3(0.48%) |
| DLNM | 31 (1.50%) | 21(1.45%) | 10 (1.61%) |
| Other | 11 (0.53%) | 6(0.41%) | 5(0.81%) |
| Multiple | 50 (2.41%) | 34(2.34%) | 16 (2.58%) |

*Abbreviations*: DLNM, distant lymph node metastasis.

**Table S2．Clinicopathological parameters for overall survival identified by univariate and multivariate analyses.**

|  | Univariate cox | | | | | | | Multivariate cox | | | | | | | |  |  |  |  |
| --- | --- | --- | --- | --- | --- | --- | --- | --- | --- | --- | --- | --- | --- | --- | --- | --- | --- | --- | --- |
| Variants | Hazard Ratio (95%CI) | | | | P-value | | | Hazard Ratio (95%CI) | | | | | *p*-value | | |  |  |  |  |
| Age |  | | | |  | | |  | | | | |  | | |  |  |  |  |
| ＜49 | 1 | | | |  | | |  | | | | |  | | |  |  |  |  |
| ≥49 | 1.352 [0.962, 1.899] | | | | 0.082 | | |  | | | | |  | | |  |  |  |  |
| Histology |  | | | |  | | |  | | | | |  | | |  |  |  |  |
| SCC | 1 | | | |  | | |  | | | | |  | | |  |  |  |  |
| AC | 1.040 [0.639, 1.694] | | | | 0.874 | | | 1.275 [0.777, 2.092] | | | | | 0.337 | | |  |  |  |  |
| ASC | 2.530 [1.361, 4.706] | | | | 0.003 | | | 2.343 [1.240, 4.426] | | | | | 0.009 | | |  |  |  |  |
| Differentiation |  | | | |  | | |  | | | | |  | | |  |  |  |  |
| Low | 1 | | | |  | | |  | | | | |  | | |  |  |  |  |
| Medium | 0.594 [0.414, 0.852] | | | | 0.005 | | | 0.728 [0.502, 1.054] | | | | | 0.092 | | |  |  |  |  |
| High | 0.134 [0.033, 0.544] | | | | 0.005 | | | 0.518 [0.121, 2.214] | | | | | 0.374 | | |  |  |  |  |
| Resection margin |  | | | |  | | |  | | | | |  | | |  |  |  |  |
| Negative | 1 | | | |  | | |  | | | | |  | | |  |  |  |  |
| Positive | 3.006 [1.922, 4.702] | | | | <0.001 | | | 1.099 [0.619, 1.952] | | | | | 0.748 | | |  |  |  |  |
| VAIN | 1.695 [1.025, 2.805] | | | | 0.04 | | | 1.305 [0.779, 2.187] | | | | | 0.312 | | |  |  |  |  |
| Lymph node metastasis | | |  | | |  | | | |  | | | |  | | | |  |  |
| Negative | 1 | | | |  | | |  | | | | |  | | |  |  |  |  |
| Positive | 4.876 [3.490, 6.812] | | | | <0.001 | | | 2.014 [1.355, 2.994] | | | | | 0.001 | | |  |  |  |  |
| Parametrium |  | | | |  | | |  | | | | |  | | |  |  |  |  |
| Negative | 1 | | | |  | | |  | | | | |  | | |  |  |  |  |
| Positive | 6.161 [4.044, 9.386] | | | | <0.001 | | | 2.489 [1.537, 4.032] | | | | | <0.001 | | |  |  |  |  |
| Tumor size |  | | | |  | | |  | | | | |  | | |  |  |  |  |
| ＜2 | 1 | | | |  | | |  | | | | |  | | |  |  |  |  |
| ＞2,≤4 | 2.561 [1.455, 4.510] | | | | 0.001 | | | 1.301 [0.716, 2.362] | | | | | 0.388 | | |  |  |  |  |
| ＞4 | 4.882 [2.780, 8.573] | | | | <0.001 | | | 2.200 [1.189, 4.068] | | | | | 0.012 | | |  |  |  |  |
| Stromal invasion |  | | | |  | | |  | | | | |  | | |  |  |  |  |
| ＜1/2 | 1 | | | |  | | |  | | | | |  | | |  |  |  |  |
| ≥1/2 | 3.889 [2.486, 6.086] | | | | <0.001 | | | 1.466 [0.872, 2.466] | | | | | 0.149 | | |  |  |  |  |
| LVSI |  | | | |  | | |  | | | | |  | | |  |  |  |  |
| Negative | 1 | | | |  | | |  | | | | |  | | |  |  |  |  |
| Positive | 3.954 [2.722, 5.745] | | | | <0.001 | | | 1.970 [1.274, 3.045] | | | | | 0.002 | | |  |  |  |  |
| Perineural involvement | | |  | | |  | | | |  | | | |  | | | |  |  |
| Negative | 1 | | | |  | | |  | | | | |  | | |  |  |  |  |
| Positive | 2.884 [1.950, 4.265] | | | | <0.001 | | | 1.245 [0.820, 1.891] | | | | | 0.304 | | |  |  |  |  |
| Vaginal invasion | |  | | | | | | |  | | |  | | | | |  | | |
| Negative | 1 | | | |  | | |  | | | | |  | | |  |  |  |  |
| Positive | 2.395 [1.693, 3.390] | | | | <0.001 | | | 1.311 [0.854, 2.014] | | | | | 0.216 | | |  |  |  |  |
| Corpus uterine invasion | | | |  | | |  | | | |  | | | |  | | | |  |
| Negative | 1 | | | |  | | |  | | | | |  | | |  |  |  |  |
| Positive | 2.680 [1.855, 3.871] | | | | <0.001 | | | 1.121 [0.748, 1.680] | | | | | 0.581 | | |  |  |  |  |

*Abbreviations*: SCC, squamous carcinoma; AC, adenocarcinoma; ASC, adenosquamous carcinoma; VAIN, vaginal intraepithelial neoplasia; LVSI, lymph-vascular space invasion.

Table S3. General clinical characteristics of the patients within each proposed RPA stage groups.

| Characteristic | RPA I (N=664) | RPA II (N=781) | RPA III (N=428) | RPA IV (N=198) | *p* |
| --- | --- | --- | --- | --- | --- |
| Age, n (%) |  |  |  |  | <0.001 |
| ＜49 | 361 (54.4%) | 350 (44.8%) | 197 (46.0%) | 74 (37.4%) |  |
| ≥49 | 303 (45.6%) | 431 (55.2%) | 231 (54.0%) | 124 (62.6%) |  |
| Histology, n (%) |  |  |  |  | <0.001 |
| AC | 120 (18.1%) | 98 (12.5%) | 42 (9.81%) | 24 (12.1%) |  |
| ASC | 18 (2.71%) | 19 (2.43%) | 15 (3.50%) | 12 (6.06%) |  |
| SCC | 526 (79.2%) | 664 (85.0%) | 371 (86.7%) | 162 (81.8%) |  |
| Differentiation, n (%) |  |  |  |  | <0.001 |
| high | 146 (22.0%) | 15 (1.92%) | 11 (2.57%) | 2 (1.01%) |  |
| low | 255 (38.4%) | 401 (51.3%) | 254 (59.3%) | 150 (75.8%) |  |
| medium | 263 (39.6%) | 365 (46.7%) | 163 (38.1%) | 46 (23.2%) |  |
| Resection margin, n (%) |  |  |  |  | <0.001 |
| Negative | 613 (92.3%) | 653 (83.6%) | 342 (79.9%) | 106 (53.5%) |  |
| Positive | 15 (2.26%) | 49 (6.27%) | 44 (10.3%) | 48 (24.2%) |  |
| VAIN | 36 (5.42%) | 79 (10.1%) | 42 (9.81%) | 44 (22.2%) |  |
| Lymph node metastasis, n (%) |  |  |  |  | <0.001 |
| Negative | 664 (100%) | 716 (91.7%) | 235 (54.9%) | 32 (16.2%) |  |
| Positive | 0 (0.00%) | 65 (8.32%) | 193 (45.1%) | 166 (83.8%) |  |
| Parametrium, n (%) |  |  |  |  | <0.001 |
| Negative | 664 (100%) | 779 (99.7%) | 399 (93.2%) | 138 (69.7%) |  |
| Positive | 0 (0.00%) | 2 (0.26%) | 29 (6.78%) | 60 (30.3%) |  |
| Tumor size, n (%) |  |  |  |  | <0.001 |
| ＞2，≤4 | 180 (27.1%) | 551 (70.6%) | 118 (27.6%) | 83 (41.9%) |  |

Table S3 (Continue). General clinical characteristics of the patients within each proposed RPA stage groups.

| ＞4 | 99 (14.9%) | 43 (5.51%) | 297 (69.4%) | 106 (53.5%) |  |
| --- | --- | --- | --- | --- | --- |
| ≤2 | 385 (58.0%) | 187 (23.9%) | 13 (3.04%) | 9 (4.55%) |  |
| Stromal invasion, n (%) |  |  |  |  | <0.001 |
| ＜1/2 | 664 (100%) | 179 (22.9%) | 23 (5.37%) | 8 (4.04%) |  |
| ≥1/2 | 0 (0.00%) | 602 (77.1%) | 405 (94.6%) | 190 (96.0%) |  |
| LVSI |  |  |  |  | <0.001 |
| Negative | 664 (100%) | 309 (39.6%) | 192 (44.9%) | 44 (22.2%) |  |
| Positive | 0 (0.00%) | 472 (60.4%) | 236 (55.1%) | 154 (77.8%) |  |
| Perineural involvement, n (%) |  |  |  |  | <0.001 |
| Negative | 659 (99.2%) | 706 (90.4%) | 373 (87.1%) | 120 (60.6%) |  |
| Positive | 5 (0.75%) | 75 (9.60%) | 55 (12.9%) | 78 (39.4%) |  |
| Vaginal invasion, n (%) |  |  |  |  | <0.001 |
| Negative | 605 (91.1%) | 629 (80.5%) | 301 (70.3%) | 119 (60.1%) |  |
| Positive | 59 (8.89%) | 152 (19.5%) | 127 (29.7%) | 79 (39.9%) |  |
| Corpus uterine invasion, n (%) |  |  |  |  | <0.001 |
| Negative | 649 (97.7%) | 693 (88.7%) | 327 (76.4%) | 121 (61.1%) |  |
| Positive | 15 (2.26%) | 88 (11.3%) | 101 (23.6%) | 77 (38.9%) |  |
| FIGO 2018, n (%) |  |  |  |  | <0.001 |
| IA1 | 152 (22.9%) | 5 (0.64%) | 0 (0.00%) | 0 (0.00%) |  |
| IA2 | 42 (6.33%) | 3 (0.38%) | 0 (0.00%) | 0 (0.00%) |  |
| IB1 | 179 (27.0%) | 146 (18.7%) | 0 (0.00%) | 0 (0.00%) |  |
| IB2 | 137 (20.6%) | 379 (48.6%) | 0 (0.00%) | 0 (0.00%) |  |
| IB3 | 61 (9.19%) | 20 (2.56%) | 115 (26.9%) | 0 (0.00%) |  |
| IIA1 | 42 (6.33%) | 143 (18.3%) | 0 (0.00%) | 0 (0.00%) |  |

Table S3 (Continue). General clinical characteristics of the patients within each proposed RPA stage groups.

| IIA2 | 51 (7.68%) | 20 (2.56%) | 120 (28.0%) | 0 (0.00%) |  |
| --- | --- | --- | --- | --- | --- |
| IIB | 0 (0.00%) | 0 (0.00%) | 0 (0.00%) | 32 (16.2%) |  |
| IIIC1p | 0 (0.00%) | 64 (8.21%) | 184 (42.9%) | 155 (78.3%) |  |
| IIIC2p | 0 (0.00%) | 0(0.00%) | 10 (2.33%) | 11 (5.56%) |  |
| T category, n (%) |  |  |  |  | <0.001 |
| T1 | 571 (86.0%) | 615 (78.7%) | 240 (56.1%) | 100 (50.5%) |  |
| T2 | 93 (14.0%) | 166 (21.3%) | 188 (43.9%) | 98 (49.5%) |  |
| N category, n (%) |  |  |  |  | <0.001 |
| N0 | 664 (100%) | 716 (91.8%) | 235 (54.8%) | 32 (16.2%) |  |
| N1 | 0 (0.00%) | 64 (8.21%) | 184 (42.9%) | 155 (78.3%) |  |
| N2 | 0 (0.00%) | 0(0.00%) | 10 (2.33%) | 11 (5.56%) |  |
| Treatment, n (%) |  |  |  |  | <0.001 |
| S | 461 (69.4%) | 82 (10.5%) | 20 (4.66%) | 9 (4.55%) |  |
| S+ NACT/ACT | 158 (23.8%) | 94 (12.1%) | 31 (7.23%) | 16 (8.08%) |  |
| S+ RT | 31 (4.67%) | 222 (28.5%) | 63 (14.7%) | 19 (9.60%) |  |
| S+ RT + NACT/ACT | 7 (1.05%) | 170 (21.8%) | 163 (38.0%) | 63 (31.8%) |  |
| S+ CCRT | 1 (0.15%) | 171 (21.9%) | 105 (24.5%) | 59 (29.8%) |  |
| S+ CCRT + NACT/ACT | 6 (0.90%) | 41 (5.26%) | 47 (11.0%) | 32 (16.2%) |  |

*Abbreviations*: SCC, squamous carcinoma; AC, adenocarcinoma; ASC, adenosquamous carcinoma; VAIN, vaginal intraepithelial neoplasia; LVSI, lymph-vascular space invasion.

Table S4．5-year overall survival and progression-free survival for proposed RPA group in training cohort and internal validation cohort.

| RPA | Training cohort | | | |  | Internal validation cohort | | | |
| --- | --- | --- | --- | --- | --- | --- | --- | --- | --- |
|  | 5-year OS | 95% CI | 5-year PFS | 95% CI |  | 5-year OS | 95% CI | 5-year PFS | 95% CI |
| I | 0.98 | (0.968,0.993) | 0.947 | (0.927,0.968) |  | 0.995 | (0.984,1.000) | 0.982 | (0.962,1.000) |
| II | 0.95 | (0.932,0.969) | 0.921 | (0.898,0.945) |  | 0.932 | (0.900,0.965) | 0.918 | (0.884,0.953) |
| III | 0.855 | (0.814,0.898) | 0.815 | (0.770,0.862) |  | 0.85 | (0.791,0.913) | 0.799 | (0.734,0.869) |
| IV | 0.642 | (0.570,0.723) | 0.607 | (0.535,0.690) |  | 0.683 | (0.547,0.852) | 0.646 | (0.511,0.817) |

Abbreviations: OS, overall survival; PFS, progression-free survival; CI, confidence interval.

Table S5. The comparisons of AUC and C-index of RPA model with the FIGO 2018/9^th^ edition T category/N category in internal validation cohort.

|  |  | OS | | | |  | | PFS | | | | | | |  | | LRRFS | | | | | |  | DMFS | | | | | |
| --- | --- | --- | --- | --- | --- | --- | --- | --- | --- | --- | --- | --- | --- | --- | --- | --- | --- | --- | --- | --- | --- | --- | --- | --- | --- | --- | --- | --- | --- |
|  |  | AUC/ C-index | | 95% CI | *p*-value | |  | | AUC/ C-index | | 95% CI | | *p*-value | |  | | AUC/ C-index | | 95% CI | | *p*-value | |  | AUC/  C-index | | 95% CI | *p*-value | | |
| AUC | RPA | 0.772 | 0.714-0.831 | | Ref |  | | 0.76 | | 0.702-0.817 | | Ref | |  | | 0.776 | | 0.709-0.843 | | Ref | |  | | 0.745 | 0.676-0.816 | | | Ref |  |
|  | FIGO 2018 | 0.704 | 0.623-0.782 | | 0.035 |  | | 0.71 | | 0.643-0.777 | | 0.164 | |  | | 0.683 | | 0.600-0.771 | | 0.024 | |  | | 0.695 | 0.613-0.777 | | | 0.23 |  |
|  | T | 0.595 | 0.522-0.667 | | <0.001 |  | | 0.59 | | 0.527-0.654 | | <0.001 | |  | | 0.574 | | 0.597-0.652 | | <0.001 | |  | | 0.575 | 0.500-0.651 | | | <0.001 |  |
|  | N | 0.679 | 0.601-0.752 | | <0.001 |  | | 0.674 | | 0.61-0.737 | | 0.009 | |  | | 0.673 | | 0.594-0.753 | | 0.001 | |  | | 0.659 | 0.582-0.735 | | | 0.024 |  |
| C-index | RPA | 0.741 | 0.687-0.795 | | Ref |  | | 0.724 | | | 0.673-0.775 | | Ref |  | | 0.745 | | 0.680-0.810 | | Ref | |  | | 0.712 | 0.650-0.774 | | | Ref |  |
|  | FIGO 2018 | 0.676 | 0.608-0.744 | | 0.047 |  | | 0.679 | | | 0.618-0.74 | | 0.13 |  | | 0.669 | | 0.589-0.749 | | 0.033 | |  | | 0.662 | 0.587-0.737 | | | 0.162 |  |
|  | T | 0.583 | 0.518-0.648 | | <0.001 |  | | 0.575 | | | 0.517-0.633 | | <0.001 |  | | 0.569 | | 0.500-0.643 | | <0.001 | |  | | 0.56 | 0.490-0.630 | | | <0.001 |  |
|  | N | 0.646 | 0.581-0.710 | | 0.002 |  | | 0.649 | | | 0.591-0.707 | | 0.011 |  | | 0.658 | | 0.583-0.733 | | 0.017 | |  | | 0.633 | 0.561-0.705 | | | 0.027 |  |

*Abbreviations*: OS, overall survival; PFS, progression-free survival; LRRFS, locoregional relapse-free survival; DMFS, distant metastasis-free survival; AUC, the area under the receiver operating characteristic curve; C-index, concordance index; CI, confidence interval.

Table S6. The comparisons of AUC and C-index of RPA model with the FIGO 2018/9th edition T category/N category in training cohort without patients received neoadjuvant chemotherapy.

|  |  | | OS | | | |  | | PFS | | | | | |  | | LRRFS | | | |  | DMFS | | | | | | |
| --- | --- | --- | --- | --- | --- | --- | --- | --- | --- | --- | --- | --- | --- | --- | --- | --- | --- | --- | --- | --- | --- | --- | --- | --- | --- | --- | --- | --- |
|  |  |  | AUC/ C-index | | 95% CI | *p*-value | |  | | AUC/ C-index | | 95% CI | *p*-value | |  | AUC/ C-index | | | 95% CI | *p*-value | | |  | | AUC/ C-index | | 95% CI | *p*-value |
| AUC | | RPA | 0.786 | 0.742-0.831 | | Ref | |  | | 0.711 | 0.664-0.758 | | | Ref |  | 0.687 | | 0.631-0.743 | | Ref | | |  | 0.745 | | 0.686-0.805 | | Ref |
|  |  | FIGO 2018 | 0.729 | 0.681-0.778 | | 0.005 | |  | | 0.666 | 0.621-0.712 | | | 0.018 |  | 0.660 | | 0.608-0.712 | | 0.230 | | |  | 0.686 | | 0.625-0.746 | | 0.012 |
|  |  | T | 0.625 | 0.576-0.675 | | <0.001 | |  | | 0.602 | 0.600-0.645 | | | <0.001 |  | 0.608 | | 0.559-0.657 | | 0.015 | | |  | 0.593 | | 0.536-0.650 | | <0.001 |
|  |  | N | 0.693 | 0.643-0.743 | | <0.001 | |  | | 0.638 | 0.595-0.681 | | | <0.001 |  | 0.631 | | 0.581-0.680 | | 0.001 | | |  | 0.661 | | 0.602-0.721 | | 0.003 |
| C-index | | RPA | 0.776 | 0.735-0.817 | | Ref | |  | | 0.706 | 0.663-0.749 | | | Ref |  | 0.681 | | 0.628-0.734 | | Ref | | |  | 0.740 | | 0.684-0.795 | | Ref |
|  |  | FIGO 2018 | 0.717 | 0.672-0.762 | | 0.002 | |  | | 0.662 | 0.621-0.703 | | | 0.006 |  | 0.653 | | 0.604-0.702 | | 0.190 | | |  | 0.685 | | 0.628-0.742 | | 0.018 |
|  |  | T | 0.623 | 0.577-0.669 | | <0.001 | |  | | 0.594 | 0.555-0.633 | | | <0.001 |  | 0.599 | | 0.553-0.645 | | 0.007 | | |  | 0.587 | | 0.533-0.641 | | <0.001 |
|  |  | N | 0.681 | 0.635-0.727 | | <0.001 | |  | | 0.633 | 0.594-0.672 | | | <0.001 |  | 0.627 | | 0.581-0.673 | | 0.007 | | |  | 0.659 | | 0.603-0.715 | | 0.002 |

*Abbreviations*: OS, overall survival; PFS, progression-free survival; LRRFS, locoregional relapse-free-survival; DMFS, distant metastasis-free survival; AUC, the area under the receiver operating characteristic curve; C-index, concordance index; CI, confidence interval.

Table S7. The comparisons of AUC and C-index of RPA model with the FIGO 2018/9^th^ edition T category/N category in internal validation cohort without patients received neoadjuvant chemotherapy.

|  |  | OS | | | |  | | PFS | | | | | | |  | | LRRFS | | | | | |  | DMFS | | | | | |
| --- | --- | --- | --- | --- | --- | --- | --- | --- | --- | --- | --- | --- | --- | --- | --- | --- | --- | --- | --- | --- | --- | --- | --- | --- | --- | --- | --- | --- | --- |
|  |  | AUC/ C-index | | 95% CI | *p*-value | |  | | AUC/ C-index | | 95% CI | | *p*-value | |  | | AUC/ C-index | | 95% CI | | *p*-value | |  | AUC/  C-index | | 95% CI | *p*-value | | |
| AUC | RPA | 0.780 | 0.717-0.844 | | Ref |  | | 0.781 | | 0.722-0.840 | | Ref | |  | | 0.776 | | 0.705-0.860 | | Ref | |  | | 0.777 | 0.714-0.841 | | | Ref |  |
|  | FIGO 2018 | 0.693 | 0.608-0.778 | | 0.024 |  | | 0.699 | | 0.623-0.775 | | 0.025 | |  | | 0.656 | | 0.556-0.757 | | 0.006 | |  | | 0.704 | 0.621-0.788 | | | 0.056 |  |
|  | T | 0.590 | 0.513-0.667 | | <0.001 |  | | 0.575 | | 0.509-0.642 | | <0.001 | |  | | 0.545 | | 0.465-0.625 | | <0.001 | |  | | 0.588 | 0.512-0.664 | | | <0.001 |  |
|  | N | 0.672 | 0.593-0.751 | | 0.003 |  | | 0.680 | | 0.610-0.750 | | 0.004 | |  | | 0.669 | | 0.580-0.759 | | 0.012 | |  | | 0.671 | 0.592-0.750 | | | 0.005 |  |
| C-index | RPA | 0.756 | 0.698-0.814 | | Ref |  | | 0.706 | | | 0.663-0.749 | | Ref |  | | 0.75 | | 0.675-0.825 | | Ref | |  | | 0.733 | 0.671-0.795 | | | Ref |  |
|  | FIGO 2018 | 0.663 | 0.589-0.737 | | 0.007 |  | | 0.662 | | | 0.621-0.703 | | 0.011 |  | | 0.643 | | 0.550-0.736 | | 0.007 | |  | | 0.671 | 0.595-0.747 | | | 0.074 |  |
|  | T | 0.573 | 0.505-0.641 | | <0.001 |  | | 0.595 | | | 0.556-0.634 | | <0.001 |  | | 0.537 | | 0.461-0.613 | | <0.001 | |  | | 0.575 | 0.503-0.647 | | | <0.001 |  |
|  | N | 0.638 | 0.568-0.708 | | <0.001 |  | | 0.633 | | | 0.594-0.672 | | <0.001 |  | | 0.655 | | 0.571-0.739 | | 0.019 | |  | | 0.641 | 0.568-0.715 | | | 0.009 |  |

*Abbreviations*: OS, overall survival; PFS, progression-free survival; LRRFS, locoregional relapse-free survival; DMFS, distant metastasis-free survival; AUC, the area under the receiver operating characteristic curve; C-index, concordance index; CI, confidence interval.

Table S8. Hazard ratios of different treatment modalities in four RPA subgroups.

| Group | Treatment | No. | HR_OS_ (95% CI) | *p* value | HR_PFS_ (95% CI) | *p* value |
| --- | --- | --- | --- | --- | --- | --- |
| RPA I | S | 461 | Reference |  | Reference |  |
|  | S+CCRT/RT±NACT/ACT | 45 | 1.473 (0.181, 11.98) | 0.717 | 0.592 (0.225, 1.558) | 0.288 |
|  | S+NACT/ACT | 158 | 1.181 (0.305, 4.575) | 0.809 | 0.436 (0.059, 3.225) | 0.416 |
| RPA II | S | 82 | Reference |  | Reference |  |
|  | S+CCRT±NACT/ACT | 211 | 0.396 (0.161, 0.976) | 0.044 | 0.422 (0.208, 0.857) | 0.017 |
|  | S+NACT/ACT | 97 | 1.084 (0.449, 2.618) | 0.857 | 0.654 (0.297, 1.441) | 0.292 |
|  | S+RT | 221 | 0.41 (0.17, 0.991) | 0.048 | 0.308 (0.145, 0.656) | 0.002 |
|  | S+RT+NACT/ACT | 169 | 0.456 (0.185, 1.124) | 0.088 | 0.412 (0.196, 0.866) | 0.019 |
| RPA III | S±RT/NACT/ACT | 114 | Reference |  | Reference |  |
|  | S+CCRT | 105 | 0.479 (0.243, 0.947) | 0.034 | 0.442 (0.237, 0.825) | 0.01 |
|  | S+CCRT+NACT/ACT | 47 | 0.667 (0.290, 1.531) | 0.339 | 0.643 (0.308, 1.34) | 0.238 |
|  | S+RT+NACT/ACT | 163 | 0.467 (0.259, 0.839) | 0.011 | 0.500 (0.298, 0.841) | 0.009 |
| RPA IV | S±RT/ NACT/ACT | 44 | Reference |  | Reference |  |
|  | S+CCRT | 59 | 0.564 (0.310, 1.028) | 0.062 | 0.633 (0.354, 1.129) | 0.121 |
|  | S+CCRT+NACT/ACT | 32 | 0.269 (0.102, 0.708) | 0.008 | 0.349 (0.150, 0.809) | 0.014 |
|  | S+RT+NACT/ACT | 63 | 0.580 (0.323, 1.041) | 0.068 | 0.609 (0.346, 1.073) | 0.086 |

*Abbreviations*: S, surgery; RT, radiotherapy; NACT, neoadjuvant chemotherapy; ACT, adjuvant chemotherapy; CCRT, concurrent chemoradiotherapy; HR, hazard ratio; OS, overall survival; PFS, progression-free survival; CI, confidence interval.


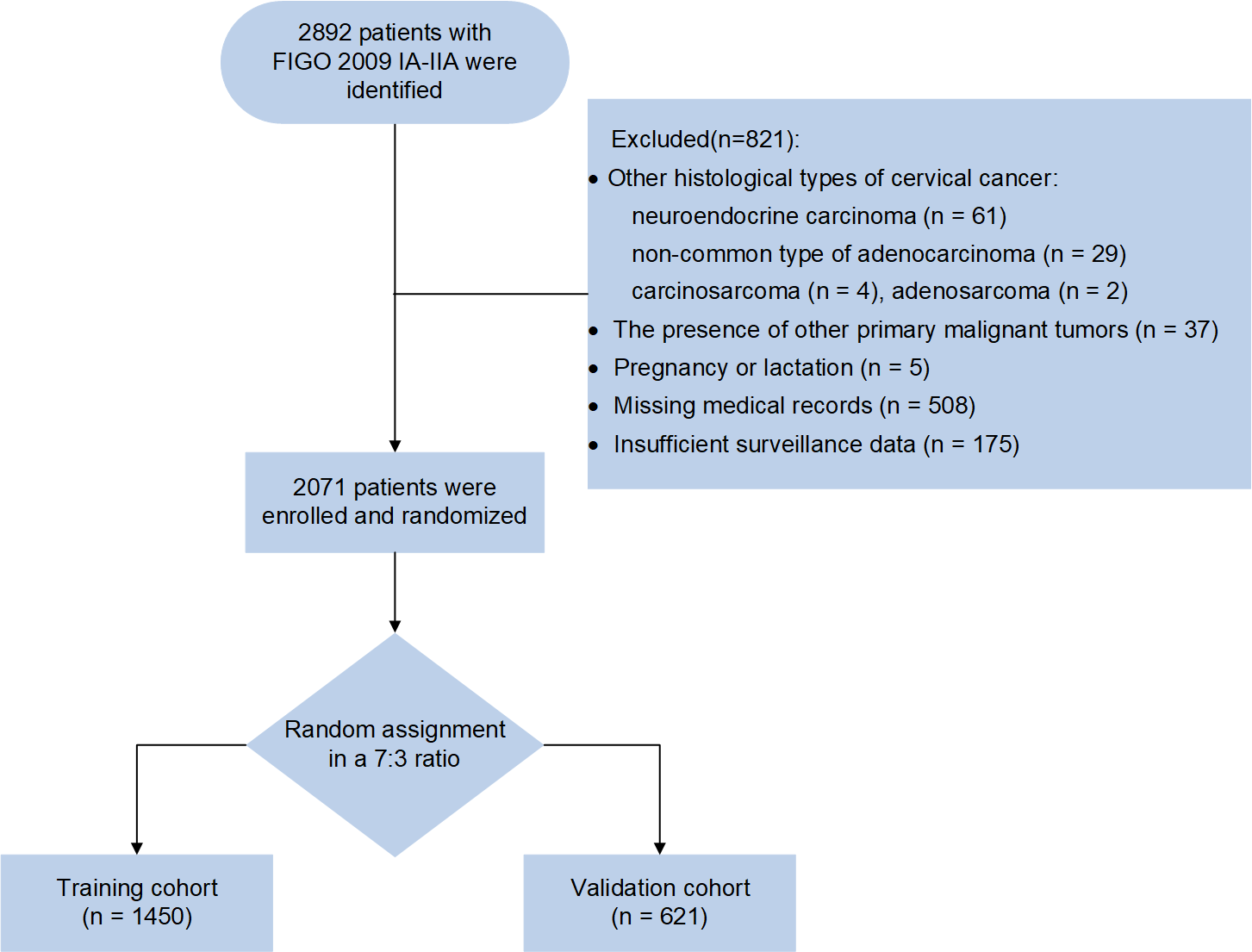


Figure S1. The flowchart of patient selection and grouping process.


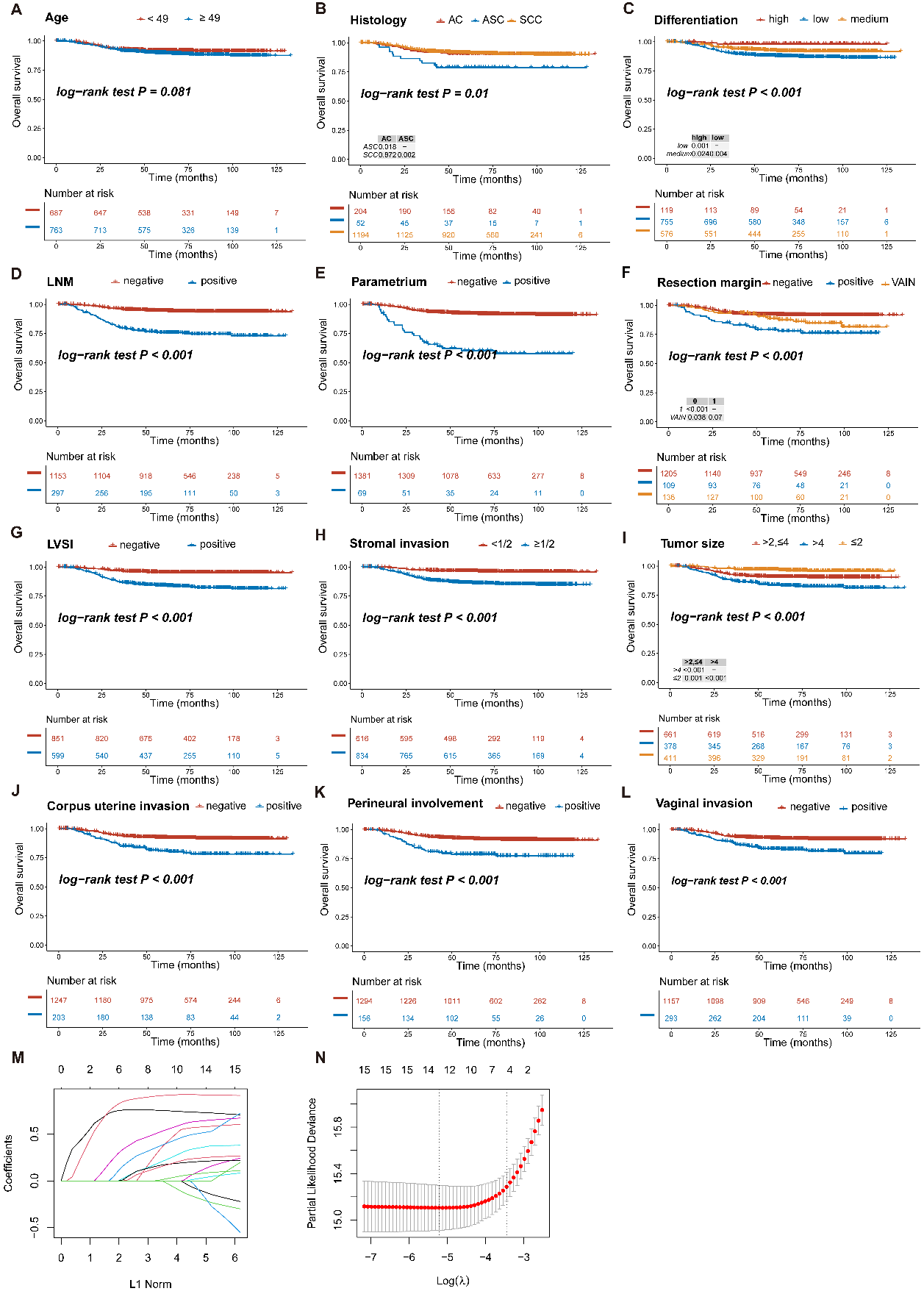


**Figure S2. Kaplan-Meier curves of overall survival for 12 clinicopathologic characteristics and factors selection using the least absolute shrinkage and selection operator (LASSO) regression analysis in the training cohort.** (A-L) Kaplan-Meier curve for histology, grade of differentiation, LNM, parametrium infiltration, resection margin, LVSI, stromal invasion, tumor size, perineural involvement, corpus uterine invasion, and vaginal invasion, respectively. (M) coefficients curves of the Factors. The different colored lines represent different variables. (N) ten-time cross validation for tuning parameter selection. The optimal value (lambda.min = 0.0054), the logarithm λ of the minimum mean square error indicated by the vertical dashed line, which will result in a model with high accuracy but complexity. Abbreviations: SCC, squamous carcinoma; AC, adenocarcinoma; ASC, adenosquamous carcinoma; LNM, lymph node metastasis; VAIN, vaginal intraepithelial neoplasia; LVSI, lymph-vascular space invasion.


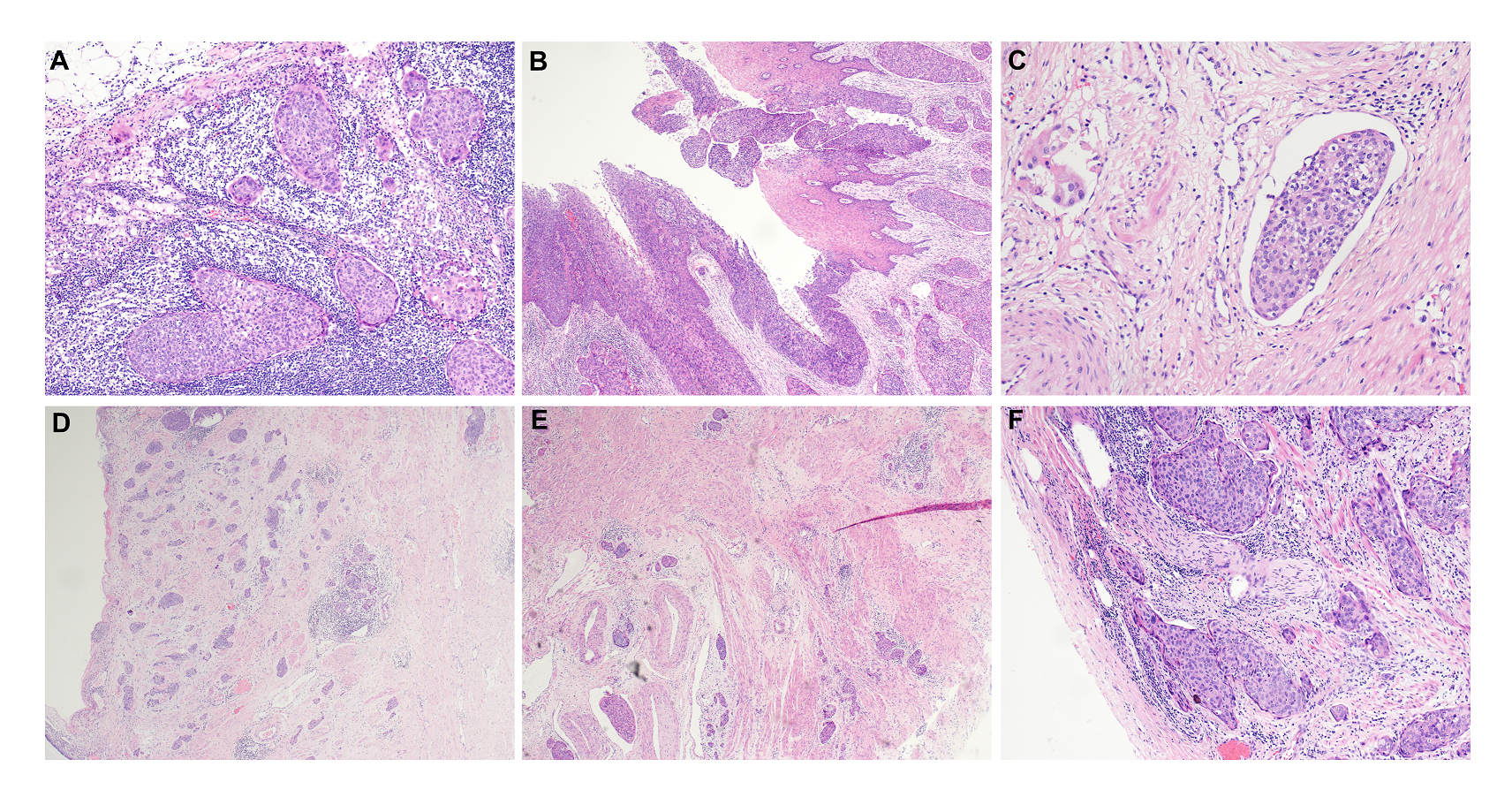
Figure S3. Histological images of relevant pathological parameters stained with H&E, 100x magnification in cervical cancer. (A) Lymph node metastasis. (B) Vaginal vault invasion. (C) Lymph-vascular space invasion. (D) Deep stromal invasion. (E) Corpus uterine invasion. (F) Perineural involvement.


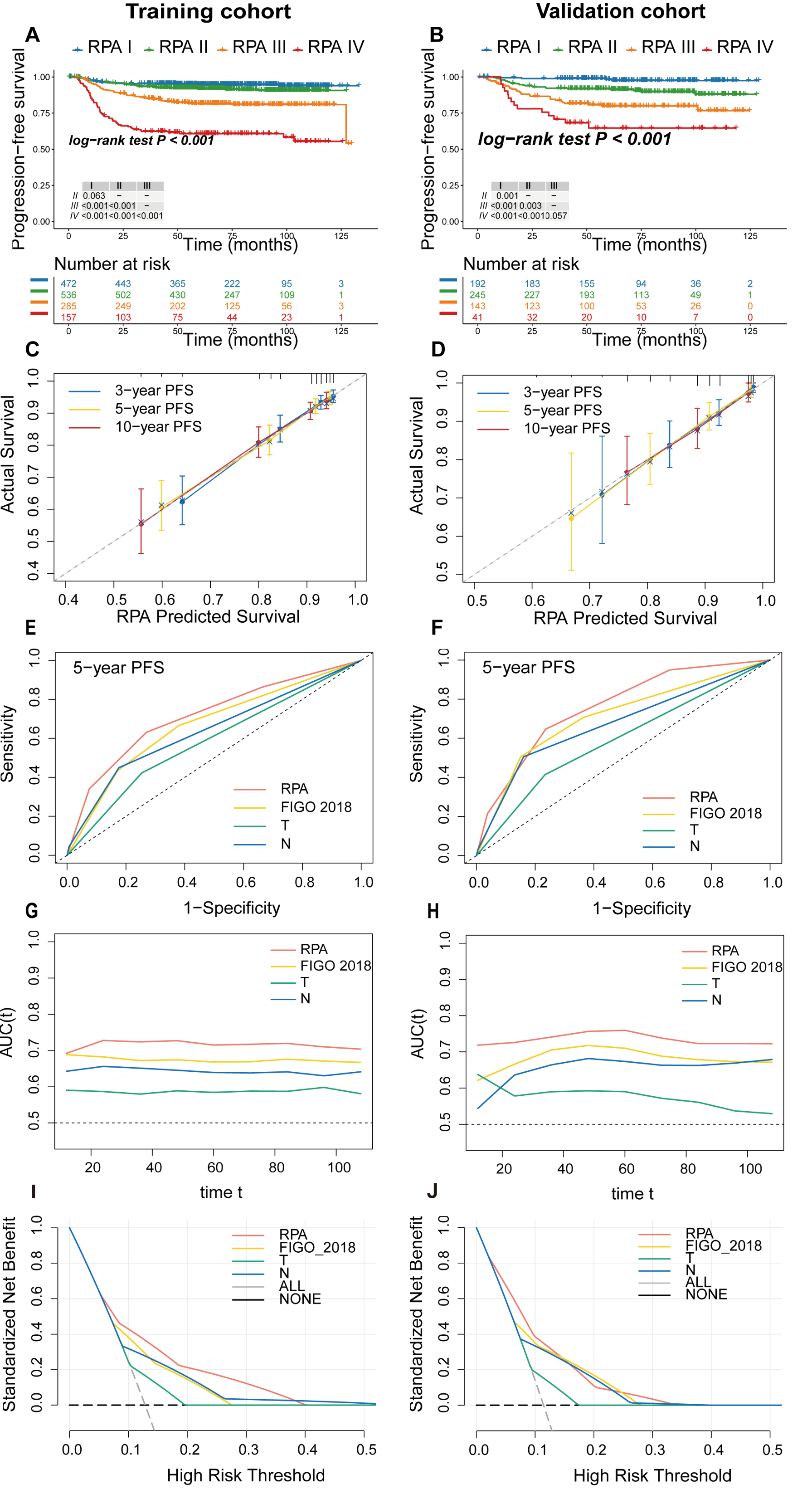


**Figure S4. Kaplan-Meier curves among RPA groups and comparison of the new RPA stage and other existing staging systems in terms of progression-free survival in training and internal validation cohorts.** (A-B) Kaplan-Meier curves for training cohort (A) and validation cohort (B). (C-D) Calibration plot predicted the 3, 5, and 10-year overall survival for training cohort (C) and validation cohort (D). (E-F) Receiver operating curves for training cohort (E) and validation cohort (F). (G-H) Time-dependent area under curves (AUC) for training cohort (G) and validation cohort (H). (I-J) Decision curve analysis for training cohort (I) and validation cohort (J). *Abbreviations*: PFS, progression -free survival. RPA, recursive partitioning analyse; AUC, the area under the receiver operating characteristic curve.


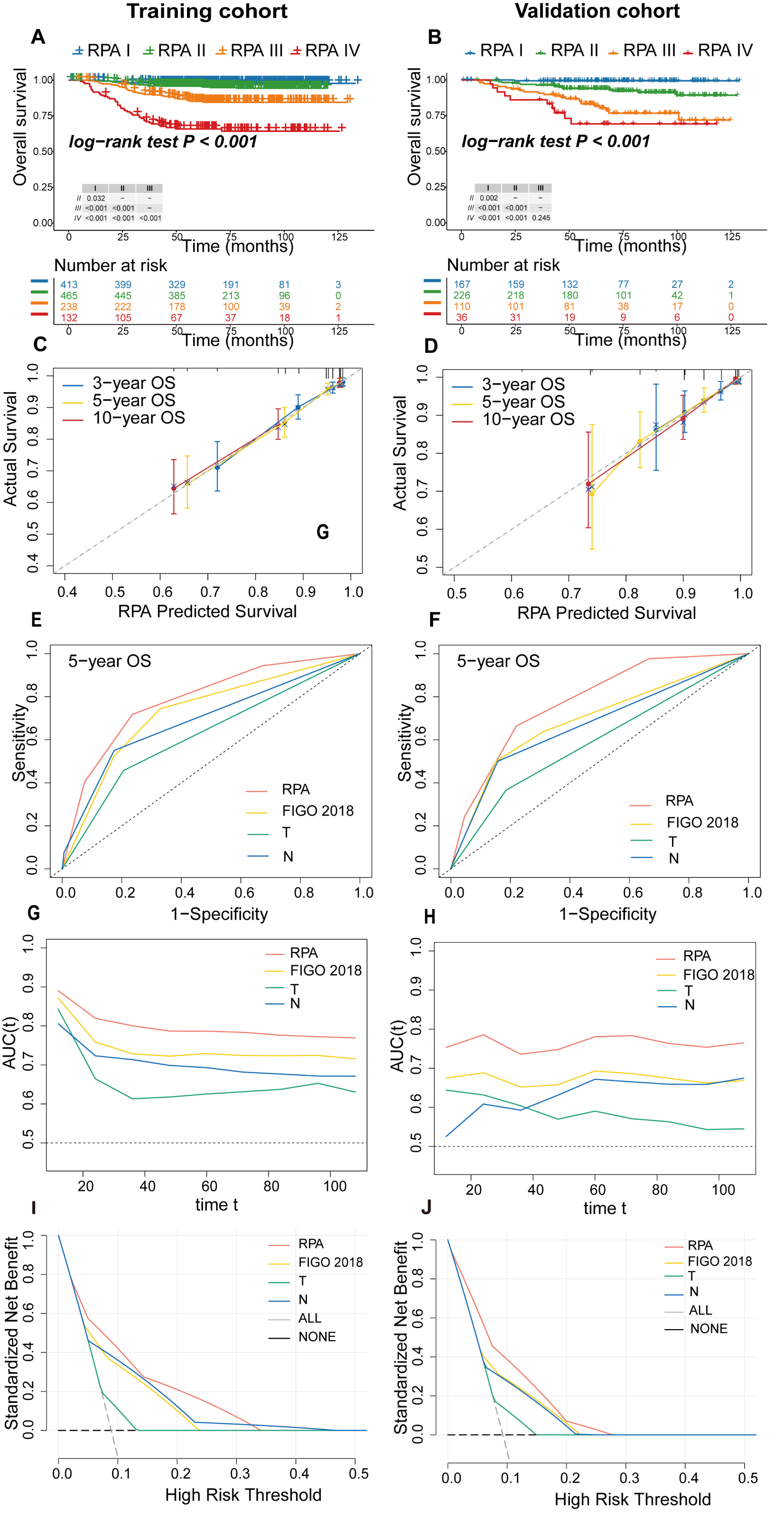


**Figure S5. Kaplan-Meier curves among RPA groups and comparison of the new RPA stage and other existing staging systems in terms of overall survival in training and internal validation cohorts without patients received neoadjuvant chemotherapy.** (A-B) Kaplan-Meier curves for training cohort (A) and validation cohort (B). (C-D) Calibration plot predicted the 3, 5, and 10-year overall survival for training cohort (C) and validation cohort (D). (E-F) Receiver operating curves for training cohort (E) and validation cohort (F). (G-H) Time-dependent area under curves (AUC) for training cohort (G) and validation cohort (H). (I-J) Decision curve analysis for training cohort (I) and validation cohort (J). *Abbreviations*: OS, overall survival. RPA, recursive partitioning analyse; AUC, the area under the receiver operating characteristic curve.


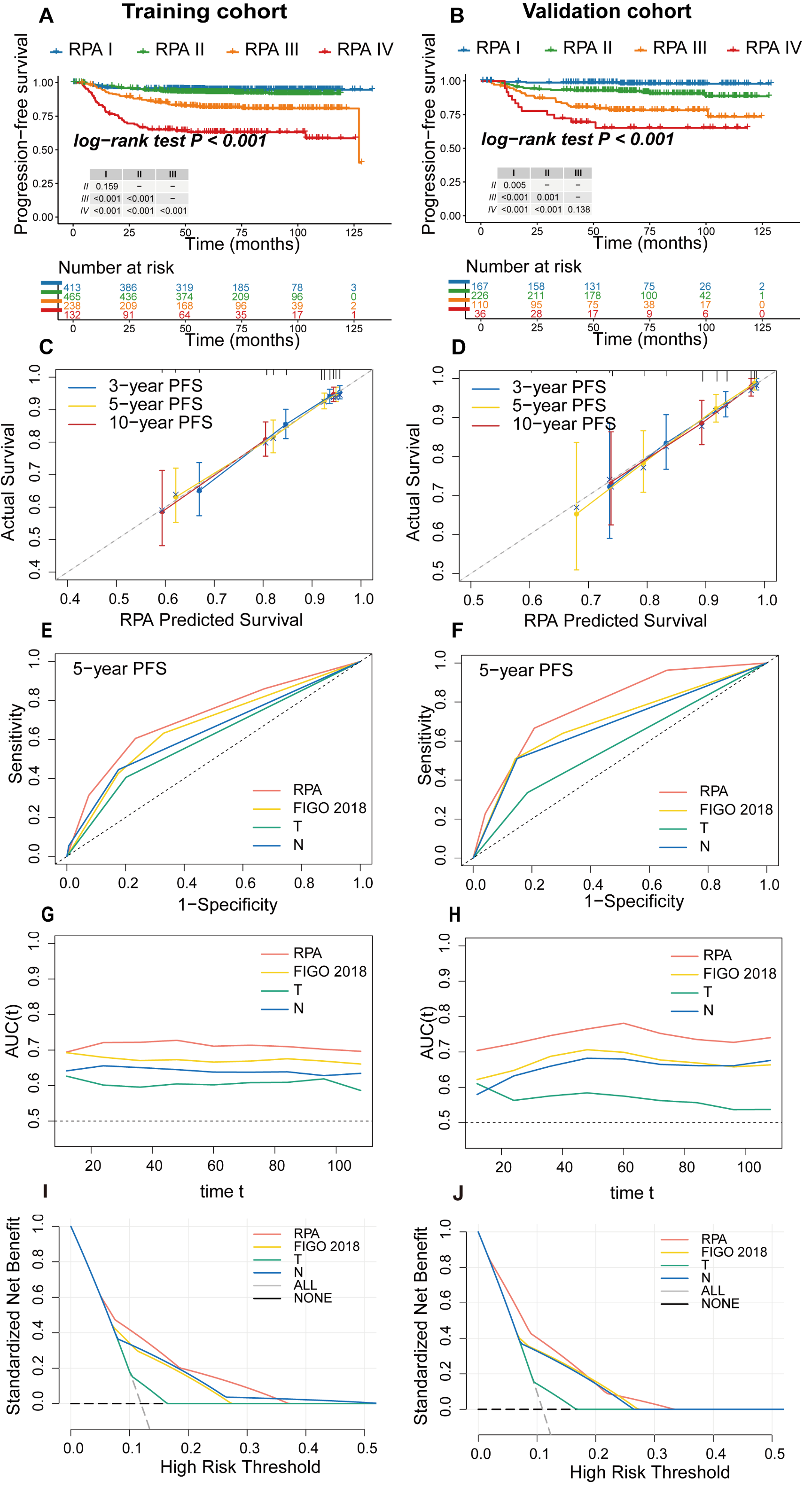


**Figure S6. Kaplan-Meier curves among RPA groups and comparison of the new RPA stage and other existing staging systems in terms of progression-free in training and internal validation cohorts without patients received neoadjuvant chemotherapy.** (A-B) Kaplan-Meier curves for training cohort (A) and validation cohort (B). (C-D) Calibration plot predicted the 3, 5, and 10-year overall survival for training cohort (C) and validation cohort (D). (E-F) Receiver operating curves for training cohort (E) and validation cohort (F). (G-H) Time-dependent area under curves (AUC) for training cohort (G) and validation cohort (H). (I-J) Decision curve analysis for training cohort (I) and validation cohort (J). *Abbreviations*: PFS, progression -free survival. RPA, recursive partitioning analyse; AUC, the area under the receiver operating characteristic curve.


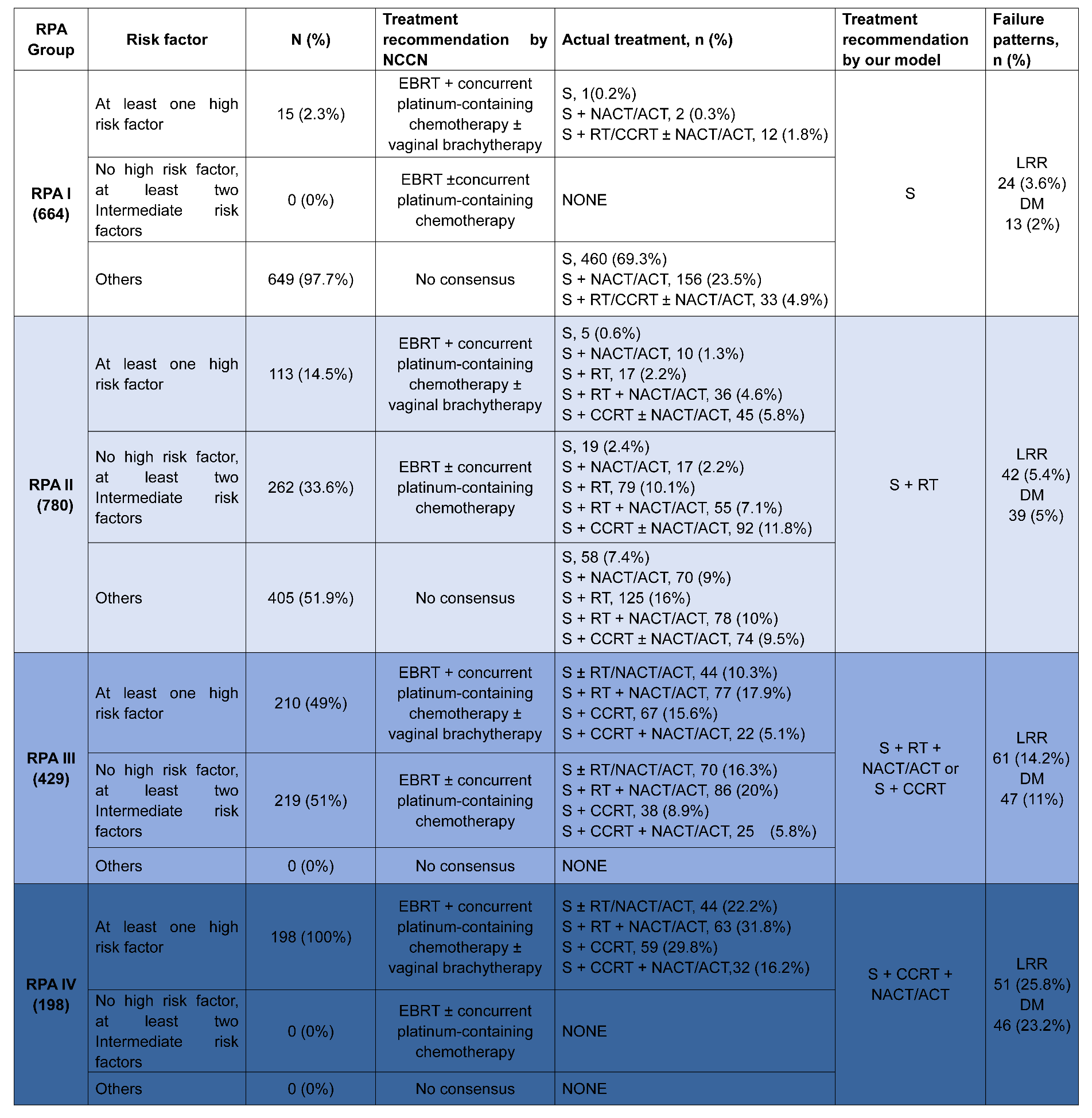


**Figure S7. Treatment recommendations and failure patterms for each RPA group.** *Abbreviations*: RPA, recursive partitioning analysis; EBRT, external beam radiation therapy S, surgery; RT, radiotherapy; NACT, Neoadjuvant chemotherapy; ACT, adjuvant chemotherapy; CCRT, concurrent chemoradiotherapy; LRR, locoregional replase; DM, distant metastasis.
